# A curtailed task for quantitative evaluation of visuomotor adaptation in the head-mounted display virtual reality environment

**DOI:** 10.3389/fpsyt.2022.963303

**Published:** 2023-02-16

**Authors:** Huiyeong Chang, Sung-Ho Woo, Sura Kang, Chan Young Lee, Jee-Young Lee, Jeh-Kwang Ryu

**Affiliations:** ^1^Interdisciplinary Program in Cognitive Science, Seoul National University, Seoul, Republic of Korea; ^2^Laboratory for Natural and Artificial Kinästhese, Convergence Research Center for Artificial Intelligence, Dongguk University, Seoul, Republic of Korea; ^3^Human Development and Rehabilitation, Graduate School of Science in Education Service, Dongguk University, Seoul, Republic of Korea; ^4^Department of Neurology, Ewha Womans University Mokdong Hospital, Ewha Womans University College of Medicine, Seoul, Republic of Korea; ^5^Department of Neurology, Seoul Metropolitan Government—Seoul National University Boramae Medical Center, Seoul National University College of Medicine, Seoul, Republic of Korea; ^6^Department of Physical Education, Dongguk University, Seoul, Republic of Korea

**Keywords:** visuomotor adaptation, HMD-based virtual reality, cerebellar ataxia, sensory prediction error, goal-directed arm movement

## Abstract

To accurately perform a goal-directed movement in continuously changing environments, it is unavoidable for individuals to adapt accordingly. The cerebellum has been known to be responsible for such process, specifically adaptation using sensorimotor information. As shown in previous studies, using HMD-VR technology in an experimental setting has similar advantages as in the real-world environment: researchers can manipulate the experimental environment, precisely control the experiments, and quantitatively analyze errors in real time. Moreover, the HMD-VR environment provides high immersiveness and embodiment which even enhance motor learning and increase engagement and motivation of individuals more than real-world environments do. In our HMD-VR-based task, the subjects were trained to adapt to a condition in which the visual information was artificially 20°clockwise rotated from the actual cursor movement. The subjects used a virtual reality tracker to move the cursor from a starting point to a target that appeared randomly at five locations, 20 cm from the starting point with an interval of 15°. Although no significant side effects were expected from experiencing the HMD-VR environment, we considered the appropriate number of trials for patients with cerebellar disease for future use in clinical settings. To examine the feasibility of our task for analysis of visuomotor adaptation pattern as shown in a real-world-based task, we created and compared two paradigms with a difference in the number of trials. As we expected, the results showed that the heading angle error decreased as the participants of both paradigms continued the task and that there was no significant difference between the two paradigms. Next, we applied our short task paradigm to patients diagnosed with cerebellar ataxia and age-matched controls for further examination of applicability to diagnosis and rehabilitation of the patients. As a result, we observed the distinguishable adaptation pattern of the patient group by using our paradigm. Overall, the results suggest that our paradigm is feasible to analyze the visuomotor adaptation pattern of healthy individuals and patients with cerebellar ataxia so may contribute to the clinical field.

## Introduction

To perform accurate movements in changing environments, humans continue to adapt to small and large variations by using sensorimotor information. The cerebellum has been known to be responsible for identifying and correcting behavioral errors for motor adaptation using sensorimotor information (1, 2). Moreover, previous studies that were conducted on patients with cerebellar ataxia using visuomotor adaptation paradigms have proved that the cerebellum is responsible for the sensorimotor adaptation process (3–5). Using the computational framework of motor control introduced by Kawato (6), researchers suggested that sensorimotor adaptation occurs by updating the forward model of the framework, which predicts the sensory outcomes of motor commands that are established based on sensory information (4, 7). If the prediction does not match the actual outcome, the adaptation process automatically assesses and corrects the errors until there is no discrepancy between predictions and observed outcomes (i.e., sensory prediction errors) (4, 7).

To explain the sensorimotor adaptation process, researchers artificially created sensory prediction errors in their experimental conditions by rotating visual information to be unmatched from the actual movement. First, prism-based studies mainly focused on adaptation to visuomotor rotation, in which participants repeated a goal-oriented arm or finger movement while wearing prism goggles that rotate the visual field (3, 8, 9). Next, technological developments allowed software-based studies to manipulate visual information in such a way that it does not match the actual hand movement by rotating the direction of a cursor movement on a computer screen instead of rotating the entire visual field (10, 11). In a software-based experimental environment, visual information (e.g., rotation angle, error feedback, etc.) can be easily and precisely manipulated, which makes it possible to examine more precisely and effectively the mechanisms underlying visuomotor adaptation (4, 7, 12). Recently, attributed to further development of technology, studies on patients with motor impairment employing HMD-VR technology were introduced (13, 14). The HMD-VR-based experimental environments allow various experimental manipulations, precise experimental control, and online quantitative analysis as in the real-world environment (15). Besides, the HMD-VR environment is well known to provide high immersiveness and embodiment which help motor learning improvements comparable to the real-world environment. These characteristics are not limited to experimental settings but may contribute to development of a diagnosis and rehabilitation environment for patients with sensorimotor adaptation impairment.

To rehabilitate impaired sensorimotor adaptation of patients with cerebellar degeneration or atrophy, many attempts have been made. For example, intensive coordination training physical therapy is provided to patients with progressive motor dysfunction due to cerebellar degeneration for improvement in motor skills and reduction in motor ataxia symptoms (16). Combined medical interventions such as physical therapy, speech therapy, and occupational therapy are provided as well to prevent secondary complications (17). Undoubtedly, HMD-VR-based tasks are applicable to rehabilitation (13, 14, 18). In a previous study using an HMD-VR-based task, the results of a self-reported questionnaire to identify side effects of experiencing an HMD-VR environment, including discomfort, fatigue, and headache, showed that no significant side effects were reported (15). This suggests that there would be no potential harm for patients to perform an HMD-VR-based task. Nevertheless, for patients who may be more vulnerable to possible side effects, it would be necessary to minimize the time of HMD-VR experience.

Before applying HMD-VR technology to a clinical setting, it is important to consider the appropriate number of trials to be given to patients. Previous neuroimaging studies revealed the involvement of the cerebellum during sensorimotor adaptation, especially during the early stage (19–21). Therefore, observation and analysis of the early stage during the adaptation process are expected to be feasible for diagnosis and rehabilitation. Moreover, to take into account the possible fatigue induced by experiencing an HMD-VR environment over a long period of time, fewer trials as possible in diagnosis and rehabilitation are necessary, especially for patients. Since very few studies have investigated visuomotor adaptation of patients with cerebellar disease in an HMD-VR environment, it is inevitable to compare tasks with a variation in the number of trials in an HMD-VR setting to consider the appropriate number of trials for diagnosis and rehabilitation of patients.

In this study, we established a visuomotor adaptation paradigm in an HMD-VR environment. The experimental task was a simple, target-directed reaching task, focused on the utilization of sensory prediction errors instead of cognitive strategies. Although simple and repetitive tasks could promote boredom, we expected HMD-VR technology to alleviate this problem as previous studies suggested that the high immersiveness and embodiment of an HMD-VR environment increase motivation and engagement of participants even in simple repetitive tasks (18, 22). However, considering our ultimate goal of applying this task to rehabilitation for patients, we even wanted to minimize boredom and fatigue by providing a task as short as possible instead of having many repetitive trials. In this sense, we made two versions of the paradigm by adjusting the number of trials within each block of adaptation. Moreover, Experiment 2 was conducted on the patients with cerebellar ataxia and age-matched controls by using the same paradigm with a shorter task procedure. Overall, we would examine the feasibility of creating a visuomotor adaptation task in the HMD-VR environment and investigate if it is applicable to diagnosis of cerebellar ataxia and rehabilitation for visuomotor adaptation.

## Materials and methods

### Participants

#### Experiment 1

A total of 30 healthy, right-handed young adults (20 males, 10 females, mean age = 22.37 ± 3.06) with normal visual acuity participated in this study. All participants had none or fewer than two virtual reality experiences and did not participate in other virtual reality experiments with similar purposes and methods. Their right-handedness was confirmed by Edinburgh Handedness Inventory (23). The Grooved Pegboard Test score of the right hand to assess their visuomotor coordination ability was within the score range for their age (24). Among them, seven participants were randomly selected to participate in a longer task while the other 23 participants participated in a shorter task. All participants gave written informed consent prior to participation. All experimental procedures, including recruitment, were performed in accordance with the ethical guidelines and procedures approved by the Dongguk University Institutional Review Board.

#### Experiment 2

A total of 29 subjects participated in the study, including 18 patients diagnosed with cerebellar ataxia (8 females, 10 males, mean age = 57.89 ± 7.31, Supplementary Table S1) and 11 healthy age-matched adults (9 females, 2 males, mean age = 51.27 ± 12.85). The patient group was recruited from individuals, who were diagnosed with spinocerebellar ataxia, recessive inherited cerebellar ataxia, idiopathic cerebellar ataxia, or spontaneous cerebellar ataxia and regularly received treatment from the motor disease clinic at the neurology department of Boramae Hospital. Any patients showing symptoms of multiple system atrophy, paraneoplastic cerebellar ataxia, autoimmune cerebellar ataxia, or acute cerebellar ataxia were excluded from recruitment. Also, patients with severe motor impairment due to underlying conditions or other serious conditions over the entire body who would not be able to perform a task were excluded from recruitment. The age-matched controls were recruited from individuals who visited the family medicine department at Boramae Hospital. They were healthy adults without neurological disease and family history of cerebellar disease. The Wisconsin Card Sorting Test (25) was conducted to evaluate the neuropsychological function of both groups. All participants gave written informed consent prior to participation. All experimental procedures, including recruitment, were performed in accordance with the ethical guidelines and procedures approved by Seoul Metropolitan Government—Seoul National University Boramae Medical Center Institutional Review Board. For data analysis of Experiment 2, data of the shorter task group from Experiment 1 was included to represent healthy young adults.

After the experiments, some participants were excluded from analysis due to their poor performances such as wandering around the target area and moving slowly toward a target. Since this study was supposed to observe and analyze the adaptation modality throughout the entire participation, the entire data of an individual had to be excluded from analysis if a single trial was poorly performed or contained an error. Overall, nine participants from the short task group in the first experiment and two participants from the patient group in the second experiment were excluded (Supplementary Table S2). Therefore, a total of 21 individuals (short: 14, long: 7) from Experiment 1 and 41 individuals (patient: 16, control: 11, young: 14) from Experiment 2 were involved in analysis.

### Experimental apparatus

**FIGURE 1:**
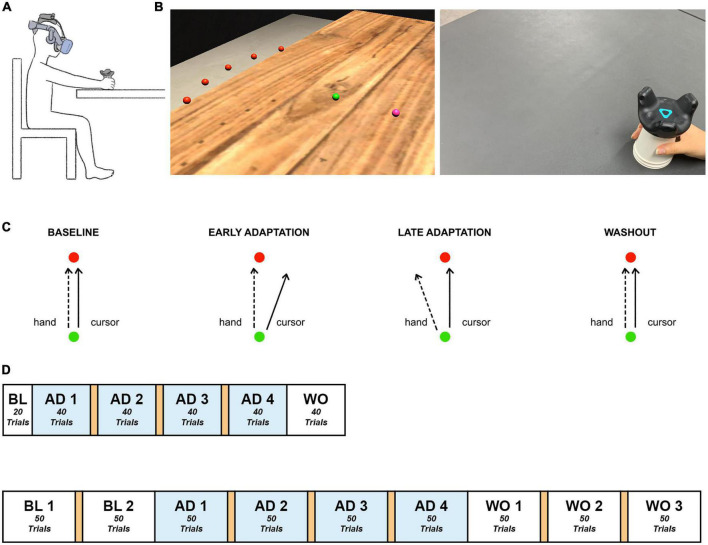
Experimental paradigm. **(A)** Experimental setup. Participants sat in front of a table, experienced the VR environment through an HMD, and moved the tracker-attached cup using their right hand. **(B)** Experimental environment. Participants made goal-directed reaching movements in the virtual reality environment. Left: The established HMD-VR experimental environment. The hand position, starting point, and target were displayed as spheres with a radius of 0.5 cm, distinguished by color (pink, green, and red, respectively), and located 12 cm above the table, which was the height of the cup. The five targets appeared at once in the practice session. Each trial of the experimental task contained one target. Each target (red) was spaced 15° apart on an invisible sector around the starting point (green) with a radius of 20 cm. Right: The real-world view. Participants moved a VR tracker attached to a cup for each reaching movement. **(C)** Experimental task. Participants moved a tracker from the starting point (green) to a target (red). Baseline: The hand trajectory and cursor trajectory were identical. Adaptation: The cursor was 20°clockwise rotated from the actual hand trajectory, and the participants learned to move their hand to compensate for the applied rotation. Washout: The visual rotation was removed, and the hand trajectory went back to the baseline state. **(D)** Experimental task flow. Top: Short task group. Bottom: Long task group. The experiment consisted of baseline, adaptation, and washout sessions. A 1-min break between blocks is indicated in orange. BL, baseline; AD, adaptation; WO, washout.

The HMD-VR experimental environment was developed using Unity 3D (Version 2019.3f1, Unity Technologies, USA). Participants were exposed to the environment using a VIVE Pro Eye Head Mounted Display (HTC, Taiwan), which has a resolution of 1,440 × 1,600 pixels per eye, and a VIVE Tracker (HTC, Taiwan) that allowed visual distortion based on real-time hand position of the participants while performing the task. A disposable paper cup was attached to the bottom of the tracker to make it easier for the participant to move the tracker by hand. The experimental environment was configured to enable the participant to sit on a chair and perform the task with the right arm on a table (Figure 1A).

### Task

Based on the visual information presented in the virtual reality experimental environment, each participant held the cup attached to the tracker with the right hand and moved it to perform a quick straight-stretch task toward the target. When the participant moved the tracker and the cursor (pink) reached the starting point (green), the target (red) appeared, which disappeared immediately upon arrival at the target position. For each trial, the target appeared randomly at a total of five positions at intervals of 15° (–30°, –15°, 0°, + 15°, + 30°) and a radius of 20 cm from the starting point (Figure 1B). The cursor, starting point, and target were all presented in the form of spheres with a radius of 0.5 cm. For the following trial, while the participants were moving the cursor back to the starting point, the cursor disappeared and appeared again at a radius of 2 cm from the starting point.

### Procedure

The experimental procedure contained three phases: baseline, adaptation, and washout. The baseline contained no visual rotation. In the adaptation phase, the visual information of the cursor movement was rotated 20°clockwise (Figure 1C). Then, the applied visual rotation disappeared in the washout phase. The short task consisted of 220 trials, 20 in the baseline phase, 160 in the adaptation phase, and 40 in the washout phase while the long task comprised 100 trials, 200 trials, and 150 trials in the three phases, respectively, so a total of 450 trials (Figure 1D). Both tasks contained 4 blocks in the adaptation phase. The long task was designed to have a similar amount of trials in adaptation and washout. The short task was adjusted to have 10 fewer trials in each adaptation block compared to the long task group. This allowed both tasks to maintain the same number of blocks in the adaptation phase but still reduced the number of trials as a whole. Since the cerebellum involvement mostly occurs in the early stage of adaptation as aforementioned in the introduction section and this study’s main focus is on the adaptation process, the short task was designed to provide only the minimum number of trials considered necessary for the analysis of the baseline and washout phases. Lastly, considering the possible fatigue induced by the experiment, a 1-min break was provided between blocks in the adaptation phase. Another break in the baseline phase and two more breaks in the washout phase were provided to the long task group, the individuals of which could experience more fatigue due to a longer participation time.

The participants practiced moving toward each target in the five locations without visual rotation. All procedures were conducted under continuous monitoring by the experimenters, and the participants were guided to perform a straightforward movement without a delay in movement from the starting point of each trial to the target.

Experiment 2 was conducted using the same experimental equipment and environment as in Experiment 1. All of the participants in Experiment 2 performed the short task (Figure 1D).

### Data analysis

**FIGURE 2:**
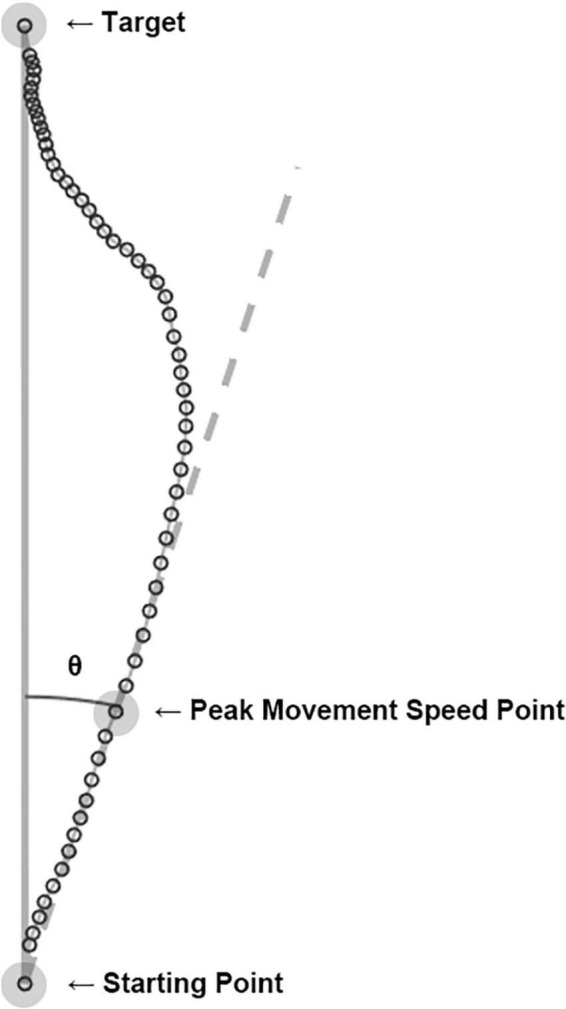
Computation of heading angle error. The cursor’s real-time positions (open circles) were connected to become a hand movement trajectory of each trial. The peak movement speed point of each trajectory was calculated, and a line (dashed line) from the starting point to the peak movement speed point represents the movement’s heading. The angle between the movement’s heading and the ideal trajectory (solid line) was computed as the heading angle error. The example shown here is an actual trial of a participant in the experiment.

MATLAB (Version 2019b, MathWorks, USA) was used for data preprocessing, smoothing, and trajectory as well as angle calculations to observe the movement characteristics associated with visual information distortion. Both MATLAB and RStudio (Version 1.3, RStudio, PBC, USA) were used for statistical analysis. The position values of the tracker were measured at 90 Hz and went through low-pass filtering with a 10 Hz cut-off frequency. The real-time position values of the tracker were used to obtain kinematic features of movement such as the movement time and peak movement speed position of each trial. The movement time was defined as the time between a departure of the cursor from the starting point and an arrival of the cursor at the target. By using the movement time and trajectory of each trial, the peak movement speed was calculated. The peak movement speed point from each trajectory would reflect the intended hand movement before the participant slowed down to correct it based on the real-time visual feedback (11). It was expected to represent the effect of sensory prediction error from a trial before. As shown in Figure 2, a straight line from the start of the movement to the peak movement speed point was drawn and compared to the ideal straight trajectory from the starting point to the target to obtain the heading angle error of each trial:


$$\theta =tan^{-1}\left(\frac{x_{n}}{z_{n}}\right)$$

within each phase (baseline, adaptation, and washout). The heading angle error was used to evaluate the visuomotor adaptation by utilizing the sensory prediction error.

To quantitatively investigate the change in task performance and the pattern of visuomotor adaptation, the heading angle error values were applied to an exponential decay function, and a non-linear least-squares model was used:


$$y=a\times e^{-t/c}$$

where *y* is the heading angle error for trial *t, a* is the starting value, and *c* is the decay constant. This exponential decay function was a modified version of a function suggested in a previous study (3). The fitted curve represented the adaptation curve or learning curve.

To compare the adaptation between the short and long task group of Experiment 1, two-tailed two-sample *t*-tests were used. For the comparison, the mean heading angle error between the short and long groups were compared. In addition, the mean of heading angle error from two exponential decay functions, one using the first 160 trials and the other using the entire 200 trials in the adaptation phase, within the long task group were compared using two-tailed two-sample *t*-tests. Normality and equal variance were checked before all *t*-tests, and *p*-values < 0.05 were considered statistically significant.

Overall, three groups performed the short task: the short task group from Experiment 1 and the two groups from Experiment 2. For further analysis, the short task group from the first experiment would be referred to as NY (normal young), the cerebellar ataxia patient group as CA (cerebellar ataxia), and the age-matched adult group as NO (normal old). To compare the adaptation among CA, NO, and NY, two additional analyses were applied. The first was one-dimensional Statistical Parametric Mapping (1D SPM) (26, 27). Since the adaptation gradually occurs over time, the change of heading angle error observed in this study can be seen as a kind of smooth time-series data. Smoothness in time-series data implies local data correlation; therefore, the number of independent processes is less, perhaps far less than the number of sampled points (28). The problem of multiple comparisons in time series data can be corrected with Bonferroni or false discovery rate (FDR) correction. However, because the Bonferroni or FDR correction is conservative, it may not be suitable for statistical inference of time series data consisting of numerous data points. In the field of functional brain imaging, the correction of multiple comparisons considering such local correlations is achieved through statistical parametric mapping (SPM) based on the random field theory (29, 30). Therefore, we also used this method to identify how the adaptation over time, which is the change in heading angle error, differs between each group. Second, through the learning curve in the form of the exponential decay described above, we identified whether the parameter corresponding to the learning rate (decay constant) was different in each subject group. In this analysis, if the cerebellum plays an important role in visuomotor adaptation, it is predicted that the learning rate of CA will be lower than that of the other two groups.

## Results

### Experiment 1: Comparison of adaptation process between the long and short task

**FIGURE 3:**
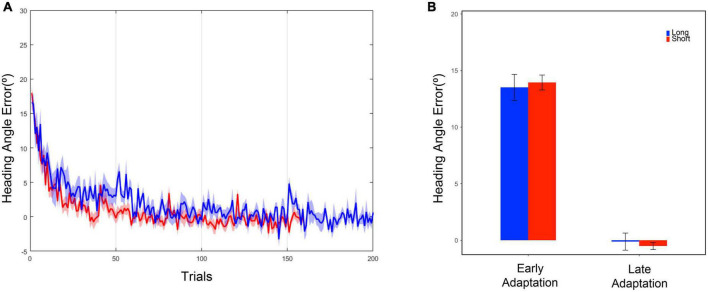
**(A)** Heading angle error curve. The blue line represents the mean, and the blue shadow represents the standard error of the mean of the long task group. The red line represents the mean, and the red shadow represents the standard error of the mean of the short task group. **(B)** Heading angle error in the early and late adaptation period. Each bar represents the mean of heading angle error, and the error bar represents the standard error of mean. The early adaptation period represents the first five trials of the adaptation phase, and the late adaptation period represents the last five trials of the adaptation phase.

Based on the results of two-tailed two-sample *t*-tests on the mean of heading angle errors in the last five trials of the baseline phase, both the long and short task group moved similarly in terms of heading angle error [*t*(19) = 0.78, *p* = 0.446] (Table 1) during the late baseline phase. When a 20° rotation was applied in the adaptation phase, the early performances of the short and long task group were similar. The performances were examined based on the results of two-tailed two-sample *t*-tests on the mean of heading angle errors from the first five trials of the adaptation phase [*t*(19) = –0.35, *p* = 0.730] (Table 1). To investigate whether the effect of the trial number existed on the performance during the adaptation phase, the performances of the short and long task group for the last five trials of the adaptation phase were compared. Two-tailed two-sample *t*-tests were used to compare the heading angle error mean. As a result, there were no significant differences between them in the late adaptation period [*t*(19) = 0.57, *p* = 0.577] (Table 1). The results suggest that there were no significant effects of the difference in the number of given trials in both the baseline and adaptation phases on the adaptation process. Overall, as the number of trials increased, the error value decreased for both groups (Figure 3).

**TABLE 1:** Movement during the three periods.

	Baseline late	Adaptation early	Adaptation late
Short	–2.24 ± 0.32	13.95 ± 0.66	–0.51 ± 0.32
Long	–1.57 ± 1.07	13.51 ± 1.16	–0.12 ± 0.76

Baseline late represents the last five trials of the baseline phase. Adaptation early represents the first five trials of the adaptation phase. Adaptation late represents the last five trials of the adaptation phase. Short and Long represent the short task group and long task group. Each value is a heading angle error (°). Mean ± standard error of mean.

For further comparison between the short and long tasks, we obtained the first 160 data points from the entire 200 data points in the adaptation phase within the long task group. The 160 points were extracted as if the participants had performed 160 trials as the short task group did. The selected 160 data points were fitted into the exponential decay function to obtain a new adaptation curve. By using the both original and new adaptation curves within the long task group, the heading angle error at the 200th trial, the last trial, was calculated. As a result of two-tailed two-sample *t*-tests, no significant difference was discovered between the original and newly calculated results [M_*original*_ = 0.23 ± 0.21, M_*new*_ = 0.17 ± 0.16, *t*(12) = 0.217, *p* = 0.832]. Through a criterion-related evidence-predictive validity test, the high predictive validity of the method measured with a small number of trials was confirmed (*r* = 0.9997). These results show that the visuomotor adaptation paradigms with 160 and 200 trials have no difference for observation and analysis of the overall adaptation pattern.

### Experiment 2: Adaptation performance of CA and healthy controls in our HMD-VR-based visuomotor adaptation task

**FIGURE 4:**
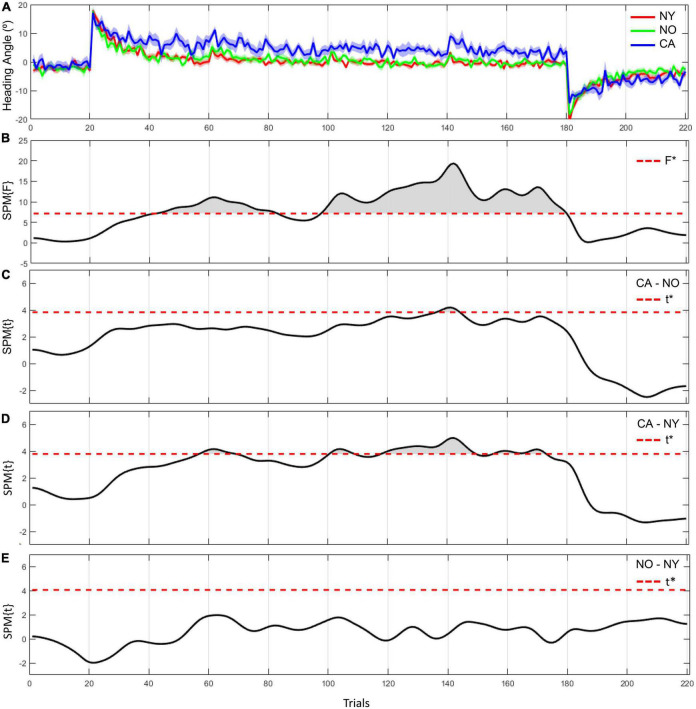
The heading angle error between the three groups and the results of statistical inference. **(A)** The red line and its shaded area represent the averaged heading angle error and standard error of mean in NY group. The green and blue lines and shaded areas represent the same in NO and CA groups, respectively. **(B–E)** F-scores in one-way ANOVA and t-scores in two-sample *t*-test as a *post hoc* paired comparison. F star (*) and t star (*) represent a significance threshold in each analysis.

In 1D SPM analysis, we observed significant differences in heading angle errors between NY, NO, and CA (Figure 4). As a result of one-way ANOVA for the heading angle errors, significant differences were observed in various trials in the adaptation period [*F*(2, 38) = 7.43, *adjusted p* < 0.05]. *Post hoc* comparisons revealed that heading angle errors in CA were significantly higher than in NY from the 57th to the 69th, the 101th to the 108th, and the 118th to the 173th trials [*t*(28) = 3.78, *Corrected p* < 0.05]. These results can be interpreted as that CA did not adapt well to a visual rotation from about the 60th trial to the second half compared to the healthy young subjects. On the other hand, in *post hoc* comparison with NO, the heading angle errors in CA were significantly higher from the 137th to the 144th trial [*t*(25) = 3.82, *Corrected p* < 0.05]. This suggests that even in comparison with NO, the difficulty of visuomotor adaptation in CA persists until the later trials. In other words, the individuals of the healthy age-matched group were able to adapt to a visual rotation as the trial continues, but patients with cerebellar ataxia had a problem with visuomotor adaptation.

**FIGURE 5:**
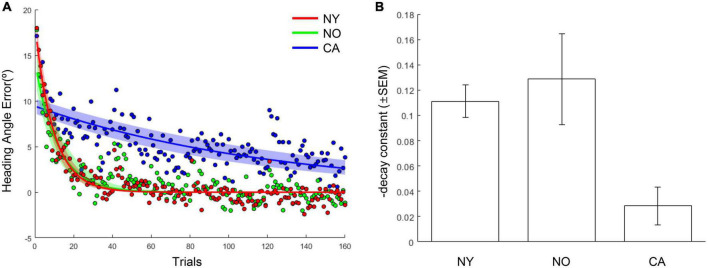
The learning curves and adaptation coefficients in NY, NO, and CA groups. **(A)** The averaged heading angles and their fitted curves in the form of exponential decay in NY, NO, and CA groups. The shaded area depicts 95% confidence intervals on fitted functions. **(B)** The averaged learning rates (decay constants) in each group. For intuitive understanding, the negative sign of decay constants was flipped to the positive sign.

The same results were consistently observed in the comparison of the adaptation coefficients of each group. As a result of fitting the averaged heading angles of each group with an exponential decay function, the adaptation coefficient of the CA group was 0.008, and the NY group was 0.103 and the NO group was 0.086 (Figure 5A). The *R*^2^ of the fitting function of the CA was 0.47 and the *R*^2^ of the NY and NO were 0.87, 0.70, respectively. To verify these observations statistically, each subject’s heading angle error was fitted with an exponential decay function, and we investigated whether the adaptation coefficients were statistically different between CA, NY, and NO with a one-way ANOVA (Figure 5B). As a result, it was confirmed that there was a significant difference between each group [*F*(2, 38) = 7.43, *p* < 0.005], and *post hoc* comparisons revealed that the adaptation coefficients of the CA were significantly lower than that of the NY [*t*(28) = 4.28, *p* < 0.001] and NO [*t*(25) = 3.00, *p* < 0.01]. These suggest that patients with cerebellar ataxia struggled in visuomotor adaptation compared to other healthy subject groups.

## Discussion

The first experiment explored the visuomotor adaptation patterns of healthy young adults in the HMD-VR environment and compared two paradigms with a difference in the number of trials. In addition to a well-known visuomotor adaptation paradigm, we utilized advantages associated with HMD-VR such as high immersiveness, embodiment, and 3D depth perception. As a result, the observed visuomotor adaptation pattern was a typical learning curve similar to that observed in the real world. Our analysis of the effect of baseline and adaptation trial number on adaptation performance between the long and short task group showed no significant difference. Moreover, the two paradigms were compared based on the exponential decay function of visuomotor adaptation. By using only 160 data points, which is the number of trials involved in the short task, we were able to obtain a similar adaptation curve as using 200 data points within the long task group. Thus, we proved that our HMD-VR-based visuomotor adaptation task with 160 trials is feasible to observe individual visuomotor adaptation.

Although it is widely accepted that human visuomotor adaptation is associated with an implicit process that utilizes sensory prediction errors, it has been suggested that explicit processes using cognitive strategies can augment the adaptation process (5, 12, 31). Therefore, it is important to examine the roles of explicit and implicit processes separately in relation to visuomotor learning. Previous studies introduced that the explicit learning process showed an immediate error reduction, while the implicit learning process showed a gradual error reduction (7, 32). These results can be clarified by the independent characteristics of the two systems; the implicit system involving sensory predictions still operated despite the fact that the explicit system using adaptation strategies was occurring (7, 12, 33). In other words, as long as the sensory prediction errors exist, the implicit system continuously updates the forward model, leading to the disappearance of the error reduction effect of the explicit learning process (7). This finding underscored that strategies-based motor learning cannot replace the implicit system during visuomotor adaptation.

Can the HMD-VR system generate an environment to effectively examine the implicit learning process? A study that compared the virtual reality environment using an HMD and the real-world environment using a computer screen suggested that HMD-VR may increase the participants’ dependence on cognitive strategies (15). In this study, however, real-time visual feedback of cursor movement for each trial was not provided to the participants, and they relied on the endpoint feedback that displayed the final position of the cursor for each trial (15). Prior research confirmed that providing real-time visual feedback on each movement induces an implicit process that utilizes sensory prediction errors (12, 34, 35). In contrast, endpoint feedback brings the adaptation process closer to cognitive strategies-based learning (12, 15, 35). Thus, cognitive dependence could be lessened in an HMD-VR environment if real-time visual feedback is provided (15). Selecting an appropriate task that can diminish cognitive dependence and establishing an experimental environment that allows the utilization of sensory prediction errors in HMD-VR are crucial. In our experiments, the cursor movements were provided as real-time visual information to examine the implicit learning process of individuals, and a simple target-reaching task was used to reduce individuals’ dependence on cognitive strategies. In addition, the heading angle error of each trial was used as a dependent variable. It was expected that the change in the outstretched hand angle as trials increased would reflect the implicit component of visuomotor adaptation, which is the utilization of sensory prediction error.

As anticipated, we observed a typical learning curve as an error reduction modality in the adaptation process. By examining the participants’ error patterns, it was possible to observe the implicit process, which shows a gradual rather than radical error decrease. More importantly, no significant difference was found between the two groups in the early and late adaptation periods. There was also no significant difference in the adaptation curve even in comparison between the 160 data points and the entire 200 data points within the long task group. Although 200 trials under the adaptation conditions would be sufficient for visuomotor adaptation, we revealed that it is still possible to analyze similarly the adaptation of individuals using fewer trials than 200 trials. Thus, we show that our short task in the HMD-VR environment is feasible for analysis of visuomotor adaptation.

This investigation of feasibility is essential especially for our ultimate goal of applying the paradigm to diagnosis and rehabilitation for patients with cerebellar disease. Although there are no significant side effects of experiencing an HMD-VR environment as suggested by a previous study (15), it is necessary to reduce the time that patients are exposed to the HMD-VR environment because they are more vulnerable to possible side effects, including fatigue. While high immersiveness and embodiment of the HMD-VR environment may improve patients’ motivation and involvement in tasks as mentioned earlier, it is also necessary to alleviate boredom caused by performing a simple and repetitive task, which is designed to reduce dependence on cognitive strategies. Moreover, the conclusion of our paradigm’s feasibility and the aforementioned results of previous studies, which is the active involvement of the cerebellum in the early stage of sensorimotor adaptation, support that the analysis of the results to be obtained through our short task is applicable to diagnosis and rehabilitation. Therefore, we applied this short task paradigm to patients to observe and analyze their visuomotor adaptation pattern.

In Experiment 2, we investigated the visuomotor adaptation pattern of three groups, such as patients with cerebellar ataxia, age-matched controls, and healthy young adults in the short task group from Experiment 1. The results showed that there was a significant difference in the overall adaptation between the patient group and the other healthy subject groups. Based on our results, we were able to conclude that the individuals of the healthy age-matched group and the healthy young group were able to adapt to a visual rotation and showed better performance as the trial continued, but the patient group showed difficulty with visuomotor adaptation. Our results support previous studies that showed that the patients with cerebellar disease had difficulty with visuomotor adaptation (3–5, 9, 36). This implies that our paradigm allows us to analyze the adaptation pattern of patients with cerebellar ataxia having difficulty in visuomotor adaptation, which is clearly distinguishable from that of young and age-matched healthy people.

Previous studies also showed that patients with cerebellar degeneration relied on cognitive strategies that were explicitly given to them to perform better and reduce errors immediately in visuomotor adaptation tasks (3, 5, 36, 37). We could relate these results to our analysis of the patients’ results and believe that the patients in our experiments performed the task without reliance on cognitive strategies. Moreover, a previous neuroimaging study showed that the cerebellum has a neural correlation with sensory prediction errors processing in reaching movements (38). In addition to the implicit learning pattern observed in our study, the finding of this previous study supports that performing our task could entail the involvement of the cerebellum, which is responsible for the utilization of sensory prediction error.

We conclude that our visuomotor adaptation task in the HMD-VR environment is feasible to observe and analyze the visuomotor adaptation pattern of individuals, is applicable to diagnosis of cerebellar ataxia, and further may be utilized in rehabilitation for visuomotor adaptation.

## Data availability statement

The raw data supporting the conclusions of this article will be made available by the authors, without undue reservation.

## Ethics statement

The studies involving human participants were reviewed and approved by the Dongguk University Institutional Review Board (202104-19) and the Seoul Metropolitan Government—Seoul National University Boramae Medical Center Institutional Review Board (30-2019-88). The participants/patients provided their written informed consent to participate in this study.

## Author contributions

J-KR and J-YL conceptualized the research. HC and J-KR designed the research. J-YL and CYL performed the clinical assessment. HC and SK performed the experiments. HC and S-HW analyzed the data and interpreted the results. HC, S-HW, and SK wrote the manuscript. J-KR supervised the research. All authors reviewed the manuscript.

## References

[1] Morton S Bastian A. Cerebellar contributions to locomotor adaptations during splitbelt treadmill walking. *J Neurosci.* (2006) 26:9107–16. 10.1523/JNEUROSCI.2622-06.2006 16957067 PMC6674518

[2] Jueptner M Weiller C. A review of differences between basal ganglia and cerebellar control of movements as revealed by functional imaging studies. *Brain.* (1998) 121:1437–49. 10.1093/brain/121.8.1437 9712006

[3] Martin T Keating J Goodkin H Bastian A Thach W. Throwing while looking through prisms: I. Focal olivocerebellar lesions impair adaptation. *Brain.* (1996) 119:1183–98. 10.1093/brain/119.4.1183 8813282

[4] Tseng Y Diedrichsen J Krakauer J Shadmehr R Bastian A. Sensory prediction errors drive cerebellum-dependent adaptation of reaching. *J Neurophysiol.* (2007) 98:54–62. 10.1152/jn.00266.2007 17507504

[5] Taylor J Klemfuss N Ivry R. An explicit strategy prevails when the cerebellum fails to compute movement errors. *Cerebellum.* (2010) 9:580–6. 10.1007/s12311-010-0201-x 20697860 PMC2996538

[6] Kawato M. Internal models for motor control and trajectory planning. *Curr Opin Neurobiol.* (1999) 9:718–27. 10.1016/s0959-4388(99)00028-8 10607637

[7] Mazzoni P Krakauer J. An implicit plan overrides an explicit strategy during visuomotor adaptation. *J Neurosci.* (2006) 26:3642–5. 10.1523/JNEUROSCI.5317-05.2006 16597717 PMC6674132

[8] Welch R Goldstein G. Prism adaptation and brain damage. *Neuropsychologia.* (1972) 10:387–94. 10.1016/0028-3932(72)90001-2 4348505

[9] Weiner M Hallett M Funkenstein H. Adaptation to lateral displacement of vision in patients with lesions of the central nervous system. *Neurology.* (1983) 33:766–72. 10.1212/wnl.33.6.766 6682520

[10] Krakauer J Ghilardi M Ghez C. Independent learning of internal models for kinematic and dynamic control of reaching. *Nat Neurosci.* (1999) 2:1026–31. 10.1038/14826 10526344

[11] Krakauer J Pine Z Ghilardi M Ghez C. Learning of visuomotor transformations for vectorial planning of reaching trajectories. *J Neurosci.* (2000) 20:8916–24. 10.1523/JNEUROSCI.20-23-08916.2000 11102502 PMC6773094

[12] Taylor J Krakauer J Ivry R. Explicit and implicit contributions to learning in a sensorimotor adaptation task. *J Neurosci.* (2014) 34:3023–32. 10.1523/JNEUROSCI.3619-13.2014 24553942 PMC3931506

[13] Huang X Naghdy F Du H Naghdy G Murray G. Design of adaptive control and virtual reality-based fine hand motion rehabilitation system and its effects in subacute stroke patients. *Comput Methods Biomech Biomed Eng.* (2018) 6:678–86. 10.1080/21681163.2017.1343687

[14] Lee S Jung H Yun S Oh B Seo H. Upper extremity rehabilitation using fully immersive virtual reality games with a head mount display: a feasibility study. *PM R.* (2020) 12:257–62. 10.1002/pmrj.12206 31218794

[15] Anglin J Sugiyama T Liew S. Visuomotor adaptation in head-mounted virtual reality versus conventional training. *Sci Rep.* (2017) 7:1–8. 10.1038/srep45469 28374808 PMC5379618

[16] Ilg W Synofzik M Brötz D Burkard S Giese M Schöls L. Intensive coordinative training improves motor performance in degenerative cerebellar disease. *Neurology.* (2009) 73:1823–30. 10.1212/WNL.0b013e3181c33adf 19864636

[17] Fonteyn E Keus S Verstappen C Schöls L de Groot I van de Warrenburg B. The effectiveness of allied health care in patients with ataxia: a systematic review. *J Neurol.* (2014) 261:251–8. 10.1007/s00415-013-6910-6 23589192

[18] Levac D Huber M Sternad D. Learning and transfer of complex motor skills in virtual reality: a perspective review. *J Neuroeng Rehabil.* (2019) 16:1–5. 10.1186/s12984-019-0587-8 31627755 PMC6798491

[19] Flament D Ellermann J Kim S Ugurbil K Ebner T. Functional magnetic resonance imaging of cerebellar activation during the learning of a visuomotor dissociation task. *Hum Brain Mapp.* (1996) 4:210–26. 10.1002/hbm.460040302 20408199

[20] Imamizu H Miyauchi S Tamada T Sasaki Y Takino R PuÈtz B Human cerebellar activity reflecting an acquired internal model of a new tool. *Nature.* (2000) 403:192–5. 10.1038/35003194 10646603

[21] Nezafat R Shadmehr R Holcomb H. Long-term adaptation to dynamics of reaching movements: a PET study. *Exp Brain Res.* (2001) 140:66–76. 10.1007/s002210100787 11500799

[22] Rizzo A Kim G. A SWOT analysis of the field of virtual reality rehabilitation and therapy. *Presence.* (2005) 14:119–46. 10.1162/1054746053967094

[23] Oldfield R. The assessment and analysis of handedness: the Edinburgh inventory. *Neuropsychologia.* (1971) 9:97–113. 10.1016/0028-3932(71)90067-4 5146491

[24] Trites R. *Neuropsychological test manual.* Ottawa, IL: Royal Ottawa Hospital (1977).

[25] Grant D Berg E. A behavioral analysis of degree of reinforcement and ease of shifting to new responses in a Weigl-type card-sorting problem. *J. Exp. Psychol.* (1948) 38:404–1. 10.1037/h0059831 18874598

[26] Pataky T. One-dimensional statistical parametric mapping in Python. *Comput Method Biomec.* (2012) 15:295–301. 10.1080/10255842.2010.527837 21756121

[27] Pataky T Robinson M Vanrenterghem J. Region-of-interest analyses of one-dimensional biomechanical trajectories: bridging 0D and 1D theory, augmenting statistical power. *PeerJ.* (2016) 4:e2652. 10.7717/peerj.2652 27833816 PMC5101620

[28] Pataky T. Generalized n-dimensional biomechanical field analysis using statistical parametric mapping. *J Biomech.* (2010) 43:1976–82. 10.1016/j.jbiomech.2010.03.008 20434726

[29] Friston K Ashburner J Kiebel S Nichols T Penny W. *Statistical parametric mapping: the analysis of functional brain images.* Amsterdam: Elsevier (2007).

[30] Friston K Holmes A Worsley K Poline J Frith C Frackowiak R. Statistical parametric maps in functional imaging: a general linear approach. *Hum Brain Mapp.* (1994) 2:189–210. 10.1002/hbm.460020402

[31] Krakauer J Mazzoni P. Human sensorimotor learning: adaptation, skill, and beyond. *Curr Opin Neurobiol.* (2011) 21:636–44. 10.1016/j.conb.2011.06.012 21764294

[32] Benson B Anguera J Seidler R. A spatial explicit strategy reduces error but interferers with sensorimotor adaptation. *J Neurophysiol.* (2011) 105:2843–51. 10.1152/jn.00002.2011 21451054 PMC3118744

[33] Shadmehr R Smith M Krakauer J. Error correction, sensory prediction, and adaptation in motor control. *Annu Rev Neurosci.* (2010) 33:89–108. 10.1146/annurev-neuro-060909-153135 20367317

[34] Izawa J Shadmehr R. Learning from sensory and reward prediction errors during motor adaptation. *PLoS Comput Biol.* (2011) 7:e1002012. 10.1371/journal.pcbi.1002012 21423711 PMC3053313

[35] Taylor J Ivry R. Flexible cognitive strategies during motor learning. *PLoS Comput Biol.* (2011) 7:e1001096. 10.1371/journal.pcbi.1001096 21390266 PMC3048379

[36] Rabe K Livne O Gizewski E Aurich V Beck A Timmann D Adaptation to visuomotor rotation and force field perturbation is correlated to different brain areas in patients with cerebellar degeneration. *J Neurophysiol.* (2009) 101:1961–71. 10.1152/jn.91069.2008 19176608

[37] Werner S Bock O Timmann D. The effect of cerebellar cortical degeneration on adaptive plasticity and movement control. *Exp Brain Res.* (2009) 193:189–96. 10.1007/s00221-008-1607-2 18949468

[38] Schlerf J Ivry R Diedrichsen J. Encoding of sensory prediction errors in the human cerebellum. *J Neurosci.* (2012) 32:4913–22. 10.1523/JNEUROSCI.4504-11.2012 22492047 PMC4332713

