# Supplementary material for: A curtailed task for quantitative evaluation of visuomotor adaptation in the head-mounted display virtual reality environment
Source: Front Psychiatry. 2023 Feb 16;13:963303. doi: 10.3389/fpsyt.2022.963303 (PMC9989973; doi:10.3389/fpsyt.2022.963303)
Supplement: Supplementary file 1 [file Table_1.docx]

Supplementary Material

Supplementary Table S1. Patient profiles

| **Subject #** | **Gender (Male/ Female)** | **Age (year)** | **Diagnosis (dx)** | **Onset (year)** | **Handedness (Right/Left)** |
| --- | --- | --- | --- | --- | --- |
| 05 | F | 58 | SCA2 | 2016 | R |
| 06 | M | 51 | ILOCA | 2018 | R |
| 07 | F | 60 | SCA3 | 2018 | R |
| 08 | F | 52 | Familial SCA | 2014 | L |
| 09 | F | 57 | SCA3 | 2012 | R |
| 10 | M | 50 | ILOCA | 2017 | R |
| 11 | M | 62 | SCA2 | 2004 | R |
| 12 | F | 74 | SCA2 | 2015 | R |
| 13 | M | 59 | ILOCA | 2014 | R |
| 14 | F | 49 | Familial SCA | 2011 | R |
| 15 | M | 61 | SCA3 | 2010 | R |
| 16 | M | 68 | ILOCA | 2018 | R |
| 17 | M | 53 | ILOCA | 2018 | R |
| 18 | M | 58 | ILOCA | 2018 | R |
| 19 | M | 46 | Familial SCA | 2019 | R |
| 20 | F | 58 | Familial SCA | 2014 | R |
| 21 | F | 69 | SCA1 (29/39+) | 2018 | R |
| 22 | M | 57 | ILOCA | 2011 | R |

Supplementary Table S2. Profiles of patients who were included in the analysis

| **Subject #** | **Gender (Male/ Female)** | **Age (year)** | **Diagnosis (dx)** | **Onset (year)** | **Handedness (Right/Left)** |
| --- | --- | --- | --- | --- | --- |
| 05 | F | 58 | SCA2 | 2016 | R |
| 06 | M | 51 | ILOCA | 2018 | R |
| 07 | F | 60 | SCA3 | 2018 | R |
| 08 | F | 52 | Familial SCA | 2014 | L |
| 09 | F | 57 | SCA3 | 2012 | R |
| 10 | M | 50 | ILOCA | 2017 | R |
| 11 | M | 62 | SCA2 | 2004 | R |
| 12 | F | 74 | SCA2 | 2015 | R |
| 14 | F | 49 | Familial SCA | 2011 | R |
| 16 | M | 68 | ILOCA | 2018 | R |
| 17 | M | 53 | ILOCA | 2018 | R |
| 18 | M | 58 | ILOCA | 2018 | R |
| 19 | M | 46 | Familial SCA | 2019 | R |
| 20 | F | 58 | Familial SCA | 2014 | R |
| 21 | F | 69 | SCA1 (29/39+) | 2018 | R |
| 22 | M | 57 | ILOCA | 2011 | R |

CA : Cerebellar Ataxia; ILOCA : Idiopathic Late Onset Cerebellar Ataxia; SCA : Spinocerebellar Ataxia
